# Rapid, real time pathology review for ECOG/ACRIN 1412: a novel and successful paradigm for future lymphoma clinical trials in the precision medicine era

**DOI:** 10.1038/s41408-018-0064-9

**Published:** 2018-02-28

**Authors:** Rebecca L. King, Grzegorz S. Nowakowski, Thomas E. Witzig, David W. Scott, Richard F. Little, Fangxin Hong, Randy D. Gascoyne, Brad S. Kahl, William R Macon

**Affiliations:** 1 Mayo Clinic, Rochester, MN USA; 2 British Columbia Cancer Agency, Vancouver, BC Canada; 3 Cancer Therapy Evaluation Program, National Cancer Institute, Bethesda, MD USA; 4 Dana-Farber Cancer Institute, Harvard T.H. Chan School of Public Health, Boston, USA; 5 Washington University School of Medicine, St. Louis, MO USA

## Abstract

ECOG/ACRIN 1412 (E1412) is a randomized, phase II open-label study of lenalidomide/RCHOP vs. RCHOP alone in adults with newly diagnosed de novo diffuse large B-cell lymphoma (DLBCL) and requires NanoString gene expression profiling (GEP) for cell-of-origin testing. Because of high ineligibility rate on retrospective expert central pathology review (ECPR), real-time (RT) ECPR was instituted to confirm diagnosis and ensure adequate tissue for GEP prior to study enrollment. Goal was notification of eligibility within 2 working days (WD). Initially, 208 patients were enrolled, 74 (35.6%) of whom were deemed ineligible by retrospective ECPR. After initiation of RT-ECPR, 219 patients were registered. Of these, 73 (33.3%) were ineligible and were declined enrollment; 47 (21.5% of total) had an ineligible diagnosis on RT-ECPR, and 26 (11.9% of total) had inadequate tissue. Because the 73 ineligible patients were never enrolled, no study slots were “lost” during this phase. Notification of eligibility occurred in an average of 1 WD (Range 0–4) with 97.3% within 2 WD. This novel RT-ECPR serves as a model for future lymphoma trials. Real-time ECPR can help to reduce costs and ensure that study slots accurately reflect the targeted population. In the precision-medicine era, rapid collection of relevant pathology/biomarker data is essential to trial success.

## Introduction

Lymphoma diagnostics has become an increasingly complex and evolving field, requiring integration of multiple testing modalities and an in-depth understanding of current World Health Organization (WHO) classification schemes, as well as National Clinical Cancer Network (NCCN) therapeutic guidelines^1–3^. In 2016, an updated revision of the WHO classification of lymphoid neoplasms was published (WHO 2016), underscoring this fact and placing emphasis on multi-modality diagnostics integrating histopathologic review, immunohistochemistry (IHC), flow cytometry, cytogenetic, and molecular genetic testing^1,2^.

In no single disease category is this paradigm shift more evident than in diffuse large B-cell lymphoma (DLBCL). The revised WHO 2017 requires the identification of germinal center B-like (GCB) and activated B-cell-like (ABC) subtypes of DLBCL. Although gene expression profiling (GEP) is required to accurately identify these, IHC algorithms will be considered acceptable since GEP is not widely available as a routine clinical test. Additionally, accurate diagnosis requires methods such as fluorescence in situ hybridization (FISH) to evaluate for rearrangements involving *MYC*, *BCL2*, and *BCL6*^1,2^. Not only do these studies allow us to better subclassify DLBCL based on its underlying biologic features, but more importantly they predict prognosis, and are increasingly important for therapy selection in the era of precision medicine^4–15^.

Although DLBCL is the most common lymphoma in the United States and other Western countries, it is still relatively rare (6.9 cases/100,000 people; ~1% of all new cancer cases) compared with non-lymphoid malignancies, and thus may pose a challenge to pathologists who do not frequently encounter these types of diseases in their practice^16–18^. In addition, pathologists at smaller centers may not have ready access to the increasingly complex array of diagnostic tools necessary to accurately evaluate lymphoma cases. Previous studies evaluating lymphoma cases sent for expert central pathology review (ECPR) support this assertion, indicating variable rates of discrepancy ranging from 5 to 25%^8,19–27^. In fact, one retrospective study evaluating rates of discrepancy on ECPR amongst all types of surgical pathology cases, suggest that lymphoma/lymph node specimens have the second highest rate of discordance at 16%^26^.

ECOG/ACRIN 1412 (E1412) is a randomized phase II open-label study of lenalidomide/RCHOP (R2CHOP) vs. RCHOP alone in patients ≥ 18 years of age with newly diagnosed de novo DLBCL. The trial statistical design is powered to determine the effects of the two treatment arms independently in GCB and ABC subtypes of DLBCL as determined by NanoString (GEP) on paraffin-embedded tumor tissue^6^. This mandated that ECPR be conducted not only to confirm the diagnosis of DLBCL and but also to ensure that adequate tissue was available for NanoString COO testing. At trial initiation, the ECPR was performed in a standard fashion, after subject randomization and after treatment had been initiated.

At approximately 50% subject accrual, data analysis indicated a high rate of patient ineligibility in addition to delays in receipt of pathology materials at the ECPR site for up to 1–2 years post-enrollment. As such, the protocol was amended to include a requirement for rapid, real-time (RT) ECPR to determine patient eligibility prior to study enrollment and randomization to therapy. ECPR included both tissue adequacy assessment for required COO testing, as well as confirmation of the submitted diagnosis. Here we present data on the 427 patients submitted for ECPR in E1412 emphasizing the importance, effectiveness, and feasibility of rapid, RT-ECPR for a DLBCL clinical trial.

## Methods

### Pathology eligibility criteria for ECOG/ACRIN 1412

For inclusion in E1412 patients were required to have a diagnosis of de novo DLBCL, based on the WHO 2008 criteria, without evidence of a concurrent low grade B-cell lymphoma. NanoString (NanoString Technologies, Seattle, WA) GEP testing by the Lymph2Cx method was required for COO subtyping and ultimate assessment of treatment outcomes by subtype^6^. Therefore, a tissue block or unstained slide sections (USS) were required as follows: 2 sections if ≥1 cm^2^ tumor; 5 sections if 0.1–1 cm^2^ tumor; 10 sections if <0.1 cm^2^ tumor^6^. Exclusionary diagnoses included: T cell/histiocyte-rich large B-cell lymphoma, primary mediastinal (thymic) large B-cell lymphoma, central nervous system DLBCL, B-cell lymphoma, unclassifiable, with features intermediate between DLBCL and Burkitt lymphoma (WHO 2008)^1^, B-cell lymphoma, unclassifiable, with features intermediate between DLBCL and classical Hodgkin lymphoma (WHO 2008)^1^, DLBCL with a concurrent low grade B-cell lymphoma or nodular lymphocyte predominant Hodgkin lymphoma, all non-DLBCL subtypes of B-cell lymphoma including follicular lymphoma, marginal zone lymphoma, mantle cell lymphoma, and B-cell lymphoma, not further classifiable.

### Pre-amendment ECPR

**Fig. 1:**
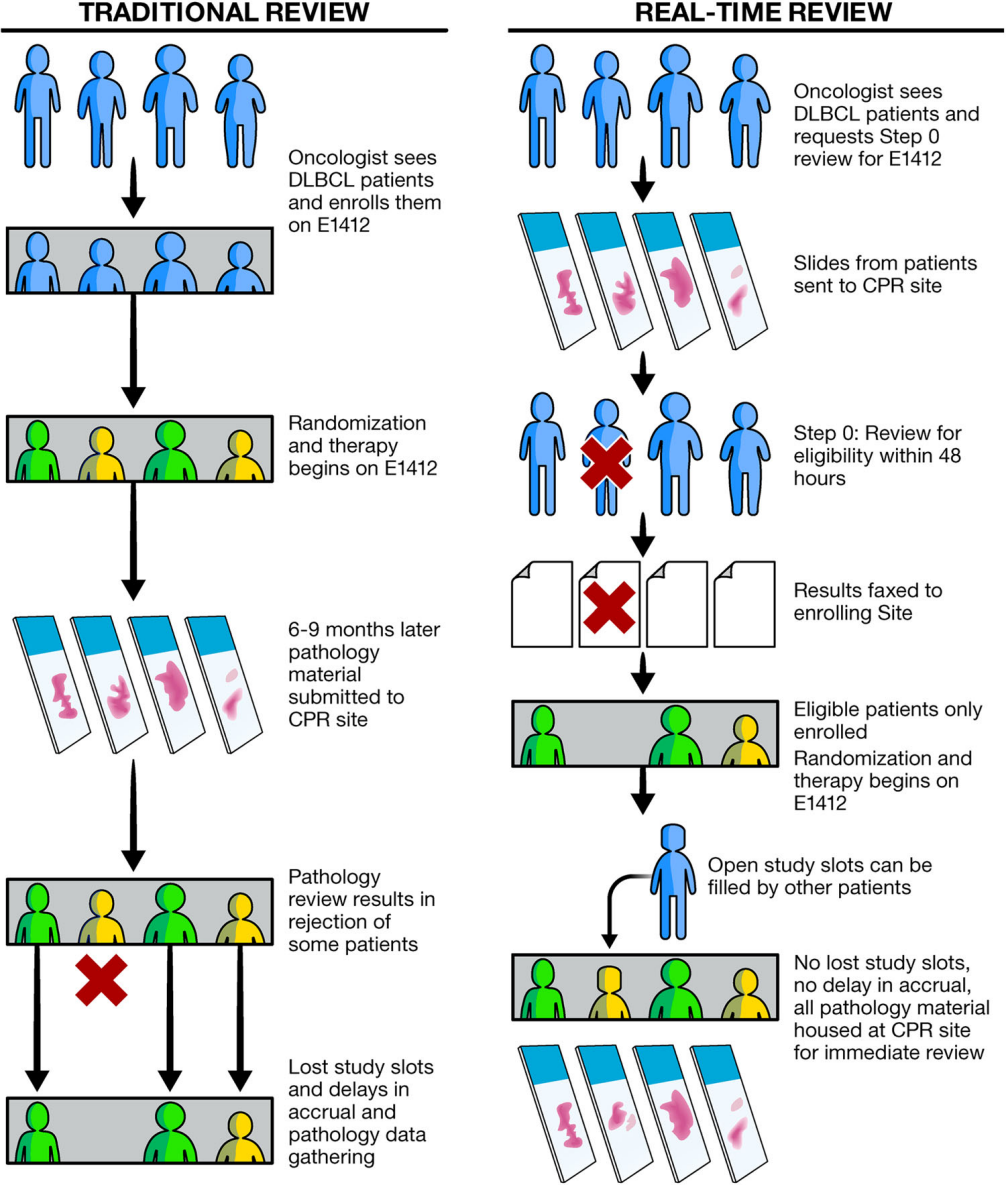
Schematic of traditional vs. real-time expert central pathology review

Patients were enrolled and assessed for eligibility based on reported diagnosis of DLBCL from the enrolling center and other clinical and laboratory features as per the study protocol. ECPR was performed retrospectively in the Division of Hematopathology, Mayo Clinic, Rochester, MN by at least one of two designated hematopathologists (RLK and WRM) as materials were received by the review site. Retrospective ECPR occurred from the date of study opening 14 May 2013 until 30 November 2015. This process is summarized in Fig. 1 (left panel).

### Post-amendment ECPR

Following protocol amendment (effective 1 December 2015 to date of study closure 17 January 2017), RT-ECPR by at least one of the two study pathologists was required prior to study enrollment. This was termed “Step 0” in the enrollment process. Details of this process are illustrated in Fig. 1 (right panel). Prior to completing enrollment of an otherwise eligible patient, enrolling sites submitted pathology materials including Hematoxylin and Eosin (H&E) stained slide of representative tumor along with a CD20 IHC slide and either a tissue block or 10 USS containing tumor. If a diagnosis of de novo DLBCL was confirmed and sufficient tissue was available for COO typing, the patient was considered eligible and the enrolling site was notified immediately via fax through a standardized “Step 0” enrollment form. Patients would then proceed with enrollment and randomization of therapy. If the diagnosis was not confirmed, or insufficient tissue remained, the enrolling site was likewise notified immediately and the patient did not proceed to enrollment on study. Protocol goal was notification within 2 working days of receipt of material at the ECPR site.

### Data collection and analysis

Date of receipt of pathology materials by a study assistant in the Division of Hematopathology at the Mayo Clinic was recorded in each case and abstracted for this study. A Step 0 form was then attached and the materials were provided to one of the two ECPR pathologists. If only a tissue block was provided, an H&E, CD20 and USS were expedited and were available to the pathologist within 24 h. In these instances, date of receipt was indicated as the first date any material was received, not the actual date of pathology review. Date of notification of the enrolling site was recorded on the “Step 0” enrollment forms.

Turnaround time (TAT) for notification of enrolling sites was calculated using the time interval (in days) between the date of receipt of materials (Day 0) and the date of notification, with exclusion of weekends. TAT for time from date of original pathology report to date of ECPR was calculated between the date the original pathology report was signed out and the date material was reviewed by a pathologist at the ECPR site, excluding weekends.

For eligible patients, USS or tissue curls were mailed from the ECPR site to a separate laboratory (British Columbia Cancer Agency, Vancouver, BC) for NanoString COO testing. Completion of COO testing was not required for assessment of study eligibility and was not performed in real-time.

Submitting diagnosis, ECPR diagnosis, eligibility status, and reason for ineligibility (if applicable) were recorded in a database for each patient. Patients were classified as either pre-amendment (retrospective review) or post-amendment (RT review).

## Results

In total, 208 patients were enrolled in E1412 prior to the study amendment over 930 days. During this phase, pathology materials were received at the ECPR site an average of 277 days following the date the original pathology report was issued (range 191–782 days). Of 208 enrolled, 74 patients (35.6%) were deemed ineligible by ECPR, either because of an ineligible diagnosis on ECPR (47, 63.5%) or inadequate tissue for the required testing/confirmation of diagnosis (27, 36.4%). When reflected as percent of total enrolled (208), 22.6% of patients had an ineligible diagnosis, and 13.0% had inadequate tissue (Table 1). Due to the retrospective nature of the ECPR during this phase, all 74 of these study slots were “lost” as patients had already been randomized and treated on study (Fig. 1). The remaining 134 patients were considered eligible for future analysis.

**Table 1:** Summary of all cases submitted to E1412. Ineligibility rate was high for reasons of tissue inadequacy as well as ineligible diagnosis on ECPR. Seventy-three study slots were preserved because of real-time ECPR

	Total submitted cases	Rejected before study enrollment “Step 0”	Total enrolled in E1412 after CPR	Total Rejected by CPR (%)	Inadequate tissue (%)	Total Ineligible diagnosis (%)	Ineligible by submitted diagnosis (%)	Ineligible by reviewed diagnosis (%)	Total pathology eligible for analysis (%)
Pre-amendment	208	0	208	74 (35.6)	27 (13.0)	47 (22.6)	7 (3.4)	40 (19.2)	134 (64.4)
Post-amendment	219	73	146	73 (33.3)	26 (11.9)	47 (21.5)	15 (6.8)	32 (14.6)	146 (66.7)
Total	427	73	354	147 (34.4)	53 (12.4)	94 (22.0)	22 (5.2)	72 (16.9)	280 (65.6)

Following amendment of the study to include RT-ECPR, 219 additional patients were submitted for Step 0 review during a 413 day period. During this phase, because of the requirement for pre-enrollment pathology review, pathology materials were received much more quickly at the ECPR site, although not always expeditiously (average 17 days, range 2–95 days). Of the 219 patients, 73 (33.3%) were ineligible and were declined enrollment either because of an ineligible diagnosis on ECPR (47, 64.4%), or inadequate tissue (26, 35.6%).When reflected as percent of total submitted (219), 21.5% of patients had an ineligible diagnosis, and 11.9% had inadequate tissue (Table 1). Because the 73 ineligible patients were never enrolled on study, no study slots were “lost” during this phase (Fig. 1).

A total of 280 patients were eligible for analysis based on ECPR for E1412. This number represents 65.6% of those submitted, and thus a rejection/ineligibility rate of 34.4%. The total rejection rate due to ECPR determination of an ineligible diagnosis was 22.0% (94 cases). The majority of these were due to discordance between the referring site and the ECPR diagnosis (72 cases), while a significant subset represented cases submitted by the referring center with an ineligible diagnosis documented on the original pathology report, with which the ECPR agreed (22 cases). Of these 22, 7 were submitted prior to the amendment, while 15 were submitted after the amendment (Table 1).

The most common ECPR diagnosis among rejected cases was DLBCL occurring with another concurrent lymphoma, usually low grade, which was not appreciated on the initial diagnostic evaluation (39 cases, 9.1% of total cases). Cases in which the submitting site diagnosed DLBCL and ECPR disagreed and diagnosed a non-DLBCL subtype of lymphoma (follicular lymphoma, marginal zone lymphoma, or B-cell lymphoma, unclassifiable) were also frequent (39, 9.1%). A summary of cases with ineligible diagnoses is shown in Table 2.

**Table 2:** Summary of cases submitted in which the diagnosis was found to be ineligible by ECPR

Exclusionary diagnoses	Total cases	Pre-amendment	Post-amendment
T cell/histiocyte-rich large B-cell lymphoma	4	1	3
B-cell lymphoma, unclassifiable, with features intermediate between DLBCL and BL^a^	8	2	6
B-cell lymphoma, unclassifiable, with features intermediate between DLBCL and classical Hodgkin lymphoma	1	1	0
DLBCL with a concurrent low-grade B-cell lymphoma or NLPHL	39	18	21
Non-DLBCL subtypes of B-cell lymphoma (FL, MZL, unclassifiable, etc.)	39	23	16
Cannot confirm lymphoma on slides	3	2	1
Total	94	47	47

^a^WHO 2008 Classification

*FL* follicular lymphoma, *MZL* marginal zone lymphoma, *NLPHL* nodular lymphocyte predominant Hodgkin lymphoma

Of 147 rejected cases, 65 (42.2%) were core/needle biopsies, 4 (2.6%) were cell blocks made from fine needle aspiration, and 78 (50.6%) were other types of excisional biopsies, punch biopsies, or resections. These numbers are similar to the overall study cohort (42.3% core/needle biopsies, 1.3% cell blocks). Among rejected cases, those with inadequate tissue had a higher proportion of core biopsies and cell blocks than those rejected for an ineligible diagnosis (71.6 vs. 24.1%, *p* < 0.05, Fisher’s exact). Rates of rejection were similar between cases reviewed initially at academic medical centers and from private hospitals/ laboratories (32.1 vs. 30.4%, *p* = 0.74, Fisher’s exact).

Post-amendment, the protocol goal was notification of enrolling sites within 2 working days and this was achieved in 97.3% of cases. When this was not achieved, it could usually be attributed to non-working holidays or cases received with only a tissue block and no stained slides for review. Average time to notification was 1 working day (range 0–4).

Although not required to be completed for enrollment, the amendment allowed for the additional GEP testing necessary for the study to be performed efficiently. All of this testing was completed on the 146 post-amendment patients within 6 weeks of receipt of their materials. Completion of this same testing for the pre-enrollment cohort took 1–2 years in some cases, due to delays in receipt of materials at the ECPR site.

## Discussion

This report is the first to detail experience and success with rapid, RT-ECPR prior to study enrollment for a multicenter National Clinical Trials Network lymphoma clinical trial. Although DLBCL represents the most common lymphoma in Western populations, the overall rate of pathology ineligibility for E1412 was high at 34.4%. This rate encompasses two major issues, neither of which is unique to this study: changes of diagnosis based on ECPR and having inadequate tissue to perform the diagnostic, prognostic and predictive studies necessary to test the hypotheses of the clinical trial.

Our results support the recent, large-scale study from France, in which expert, RT central pathology review of lymphomas was performed on over 42,000 patients between 2010 and 2013^27^. The overall rate of diagnostic change between referral and expert review in the French study was 19.7%, similar to our rate of 16.9%^27^. As in the US, DLBCL represents the most common lymphoma in their population, and accordingly was the most common lymphoma in their cohort at approximately one-third of total cases reviewed. Other studies have noted similar findings, with a discordance rate of up to 25.8% after expert review for DLBCL cases (one of the highest rates among the lymphoma types studied) in a London Regional Cancer network^19^. In fact, when comparing discordance rates amongst different specimen types in a US referral practice, rates of lymph node/lymphoma discordance after expert review were 16%, second only to gastrointestinal specimens, and higher than others including gynecologic, breast, bone/soft tissue, pulmonary, and others^26^. While some recent US-based clinical trials have shown lower rates of rejection due to ineligible diagnosis, their inclusion criteria and tissue requirements differed from those of E1412 in various ways, and thus cannot be directly compared^28–33^. The results of our study, as well as these others, highlight that, despite its prevalence, the diagnosis of DLBCL is challenging for pathologists and prone to misdiagnosis.

In the clinical trial setting such as this one, enrollment of patients with diagnoses that are ineligible can complicate data analysis leading to loss of study power to evaluate the planned endpoints. Specifically, as more DLBCL clinical trials require COO analysis to assess treatment efficacy in different subgroups, the ability to assess in real time not only the diagnosis, but certain pathologic features, as well as the adequacy of tissue for molecular genetic testing is critical. In addition, when a clinical trial is written with a specific subgroup of patients in mind (for example de novo DLBCL), and with certain exclusionary diagnoses, it is critical that CPR be performed to ensure that the enrolled population accurately reflects the study as written. Although current standard therapy may be similar for two diseases, that does not necessarily indicate that they should be included as one entity in a clinical trial setting. For example, although T cell/histiocyte-rich large B cell lymphoma has a similar standard therapy to DLBCL, NOS, these two represent pathologically distinct disease processes and in a research setting should be treated as distinct entities in order to assess for potential therapeutic advances in each disease individually.

The impact to patients of mis-classification of lymphoma which this study and others have highlighted is important outside the clinical trial realm, as well. While many of the diagnostic revisions identified in this study would likely have minimal impact on routine patient care (for example identification of a concurrent low grade lymphoma in the background of DLBCL), a significant percentage of our revisions (39/94, 41.5%) were cases submitted as DLBCL that in fact had only low grade lymphoma or lymphoma that was unclassifiable based on the tissue submitted. Changes such as these would have significant impact on therapy selection, as these patients would typically not require R-CHOP chemotherapy routinely used for DLBCL, but would be treated with less aggressive regimens. Furthermore, patients revised to a diagnosis of B-cell lymphoma, unclassifiable, with features intermediate between DLBCL and BL (WHO 2008), would be expected to frequently receive escalated therapy in accordance with a more aggressive disease process.

While this study did not directly evaluate the clinical impact of diagnostic revision, Laurent et al. reported a 17.4% overall rate of diagnostic change with potential impact on patient care^27^. Similarly, Proctor et al. reported that 11% of cases with discordant lymphoma diagnoses on secondary review would have significant change in clinical management. Furthermore, they reported that 39% would have a minor impact on patient care, and the remaining 50% would have resulted in delayed or inappropriate therapy without the secondary review^25^. Finally similar results were presented in a study by Bowen et al, evaluating review of referred lymphoma diagnoses at a single academic center. In their study, 12.9% of reviewed cases had a major diagnostic change resulting in therapeutic change following secondary review. Interestingly, their study specifically evaluated diagnostic revision from academic vs. non-academic centers, and found higher rates of discordance from non-academic centers. In contrast, perhaps surprisingly, our study did not find such a difference (academic 32.1% vs. non-academic 30.4%, *p* = 0.74, Fisher’s exact). It is uncertain what accounts for this difference, although we speculate that the line between academic and non-academic practice has become increasingly blurred in recent years as larger centers acquire smaller community-based hospitals which may then carry the larger academic name, but still operate as a small, community practice.

The WHO 2016 update now requires pathologists to perform either IHC or molecular testing in every case of DLBCL to determine COO^2^. Given the challenges of standardizing IHC testing, and lack of access to newer molecular methods, reporting of COO may be prone to discordance between practices^34^. Additionally, with the inclusion of the entity “high grade B-cell lymphoma, with *MYC* and *BCL2* or *BCL6* rearrangements” in the WHO 2016 lymphoma classification, it also becomes critical to perform FISH to exclude a *MYC* gene rearrangement in cases of lymphoma with DLBCL morphology^2^. In spite of recommendations by the WHO, this testing is likely to be variably adopted amongst pathologists, both for reasons of access to testing and knowledge of the classification changes^34^. Although E1412 did not require COO determination or *MYC* FISH in RT to initiate therapy, having these data up-front is likely to be critical for future DLBCL trials, further underscoring the need for rapid, RT-ECPR and testing in the era of targeted therapy and precision medicine.

The problem of tissue inadequacy is not unique to clinical trials, and is an unfortunately frequent occurrence with the increasing prevalence of core needle biopsy specimens and the concurrent rise in molecular/genetic testing in diagnostic pathology. Almost half of all patients submitted for enrollment on E1412 were diagnosed with lymphoma on core biopsy. While in most cases adequate for diagnosis in this study and others^35–37^, these small specimens may leave little tissue remaining for necessary molecular/genetic studies. Accordingly, both the WHO and NCCN caution strongly against using fine needle aspirate and core biopsies for the initial diagnosis of malignant lymphoma^2,3^. The high ineligibility rate due to tissue inadequacy in this study emphasizes the continuing need for larger, preferably excisional, biopsies in the diagnosis of lymphoma. Not only will this ensure sufficient tissue for possible clinical trial enrollment, but it ensures adequacy for molecular and genetic tests which are becoming increasingly required for appropriate diagnosis in the routine clinical setting both in academic and community centers.

Within the realm of clinical trials, our process of rapid, RT-ECPR provides a viable solution for the issues caused by misdiagnosis and misclassification of lymphomas. Prior to the study modification, over 1/3 of study slots were held by patients who would later become ineligible for the trial. After institution of RT-ECPR, these study slots were preserved, along with all of the costs of treating those patients on study, as patients whose pathology was ineligible were never enrolled on the trial. This results in savings not only of money and time spent treating these patients on study, but also may enable those patients to more efficiently receive appropriate therapy at their home institution.

As obtaining up-front relevant pathology and biomarker data becomes increasingly important for structuring and enrolling patients on clinical trials, cooperation between pathologists and clinicians will be critical. Increasingly, DLBCL trials are focusing on therapies directed at either GCB or ABC patients, or those with or without MYC rearrangements^8,7,9,11,14^. As this study illustrates, RT-ECPR is vital for ensuring an appropriate study population, and for collection of such data.

While promising, RT-ECPR is not without its limitations. There is no question that time is added prior to enrollment with ECPR, even when done efficiently. To minimize this, referring centers were encouraged to send their stained pathology materials, to avoid the additional time needed to cut and stain slides at the ECPR site. Additionally, the ECPR pathologists ensured that at least one pathologist was available every weekday for incoming cases. Weekends and holidays, however, do result in some inevitable delays. Bias within clinical trials is already a concern, as they may select for patients whose disease is more stable to allow time for enrollment^8^. To alleviate these concerns, some recent studies, including E1412, have allowed for up-front treatment with corticosteroids as a bridge to beginning therapy on study. E1412 allows up to 7 days pre-treatment with prednisone, as clinically indicated, without requiring a washout period^8^. As our data clearly illustrates, however, most of the pre-trial time occurred between the time that the diagnosis was made by the home institution, and the time the material was submitted for enrollment to E1412, rather than the time between submission for enrollment and notification of eligibility.

In summary, the success of this rapid, RT-ECPR for E1412 exemplifies a new era in DLBCL clinical trials and serves as a model for other lymphoma trials. With increasing complexity of lymphoma diagnostics, there is a need for central, uniform pathology review to ensure that the study cohort accurately represents the population of interest. Furthermore, RT-ECPR allows for rapid and organized collection of relevant pathology and biomarker data essential to trial success.
